# Swine acute diarrhea syndrome coronavirus-induced apoptosis is caspase- and cyclophilin D- dependent

**DOI:** 10.1080/22221751.2020.1722758

**Published:** 2020-02-24

**Authors:** Jiyu Zhang, Yuru Han, Hongyan Shi, Jianfei Chen, Xin Zhang, Xiaobo Wang, Ling Zhou, Jianbo Liu, Jialin Zhang, Zhaoyang Ji, Zhaoyang Jing, Jingyun Ma, Da Shi, Li Feng

**Affiliations:** a State Key Laboratory of Veterinary Biotechnology, Harbin Veterinary Research Institute, Chinese Academy of Agricultural Sciences, Harbin, China; b College of Animal Science, South China Agricultural University, Guangzhou, China

**Keywords:** SADS-CoV, apoptosis, apoptosis-inducing factor, pathogenesis

## Abstract

Swine acute diarrhea syndrome coronavirus (SADS-CoV), a newly discovered enteric coronavirus, is the aetiological agent that causes severe clinical diarrhea and intestinal pathological damage in piglets. To understand the effect of SADS-CoV on host cells, we characterized the apoptotic pathways and elucidated mechanisms underlying the process of apoptotic cell death after SADS-CoV infection. SADS-CoV-infected cells showed evidence of apoptosis *in vitro* and *in vivo*. The use of a pan-caspase inhibitor resulted in the inhibition of SADS-CoV-induced apoptosis and reduction in SADS-CoV replication, suggestive of the association of a caspase-dependent pathway. Furthermore, SADS-CoV infection activated the initiators caspase-8 and -9 and upregulated FasL and Bid cleavage, demonstrating a crosstalk between the extrinsic and intrinsic pathways. However, the proapoptotic proteins Bax and Cytochrome c (Cyt c) relocalized to the mitochondria and cytoplasm, respectively, after infection by SADS-CoV. Moreover, Vero E6 and IPI-2I cells treated with cyclosporin A (CsA), an inhibitor of mitochondrial permeability transition pore (MPTP) opening, were completely protected from SADS-CoV-induced apoptosis and viral replication, suggesting the involvement of cyclophilin D (CypD) in these processes. Altogether, our results indicate that caspase-dependent FasL (extrinsic)- and mitochondria (intrinsic)- mediated apoptotic pathways play a central role in SADS-CoV-induced apoptosis that facilitates viral replication. In summary, these findings demonstrate mechanisms by which SADS-CoV induces apoptosis and improve our understanding of SADS-CoV pathogenesis.

## Introduction

Apoptosis is a tightly regulated mechanism of cell death that is triggered by various extracellular (extrinsic) or intracellular (intrinsic) stimuli and modulated by anti- and pro-apoptosis cellular factors [1]. Cells undergoing apoptosis are accompanied by characteristic morphological changes, including rounding up of cells, chromatin condensation, nuclear fragmentation, and plasma membrane blebbing [2]. Apoptosis, a tightly controlled physiological process, plays crucial roles not only in the normal development and homeostasis of multicellular organisms [3,4], but also in the pathogenesis of several viral infections [5,6]. The central players in apoptosis are a family of cysteine-dependent aspartate-directed proteinases termed caspases, which catalyze key steps in the death pathway by cleavage of substrates at specific sites that contain the amino acid aspartic acid [7]. Nuclear condensation is the consequence of DNA fragmentation as manifested by the characteristic oligo nucleosome-sized DNA ladder and is mediated by the activation of a caspase-dependent endonuclease, the DNA fragmentation factor [7]. Apoptosis also represents an important antivirus defense mechanism of host cells, and viruses have evolved various sophisticated strategies to manipulate host cells such as limit anti-virus responses of the host, which may be advantageous for viral replication and persistent infection [5]. In recent years, many viruses from different families, including some coronaviruses, have been found to induce apoptosis during their infection cycles [8–12].

Coronaviruses are the largest RNA viruses identified to date. These have a positive-sense, single-stranded RNA genome of 27 − 30 kb in length that typically contains four structural proteins, namely the spike (S), nucleocapsid (N), membrane (M), and envelope (E). Coronaviruses also encode several nonstructural proteins by subgenomic mRNAs and two large polyproteins by mRNA 1. The two polyproteins are processed by viral proteinases to generate more than 10 mature cleavage products [13]. Coronaviruses cause a wide spectrum of diseases in humans and animals but primarily infect the respiratory and gastrointestinal mucosa [14]. Swine acute diarrhea syndrome coronavirus (SADS-CoV) is a recently discovered coronavirus that causes severe and acute diarrhea and rapid weight loss in piglets less than six days old. From January to May 2017, the outbreak of SADS-CoV led to the death of almost 25,000 piglets in southern China and resulted in significant economic losses [15]. After adaptation to a cell culture system (e.g. Vero E6), SADS-CoV undergoes a cytolytic life cycle. A hallmark of SADS-CoV infection of cultured cells is the formation of syncytial cells, which rapidly spread from virus-infected cells to the surrounding cells. The syncytia are subsequently destroyed, and the cells round up and detach from the substratum, concomitant with the release of virions. However, the nature of the events leading to cell death in SADS-CoV-infected cells remains largely unknown.

To date, whether SADS-CoV triggers apoptotic cell death and virus-induced apoptosis aids or worsens viral replication and pathogenicity remain unclear. Therefore, the present study aimed to investigate the *in vitro* and *in vivo* effects of SADS-CoV infection and their underlying molecular mechanisms to improve our understanding of the correlation between SADS-CoV-induced apoptosis and its pathogenic mechanisms.

## Material and methods

### Cells, viruses, reagents, and antibodies

Vero E6 cells and IPI-2I cells (porcine intestinal epithelial cells) were cultured in Dulbecco’s Minimal Essential Medium (DMEM; Invitrogen) with 10% fetal bovine serum (FBS; Invitrogen) and antibiotic-antimycotic solutions (100×; Invitrogen). The cells were maintained at 37°C in a humidified 5% CO_2_ incubator. The SADS-CoV was isolated from intestinal tract contents of SADS-CoV-infected piglets in Guangdong Province, China, and identified by physicochemical and neutralization testing and RT–PCR and sequence analyses [15]. SADS-CoV propagated in Vero E6 cells and virus titers was determined by 50% tissue culture infective doses (TCID_50_) as previously described [16]. Z-VAD-FMK (R&D Systems), Z-IETD-FMK (BD Pharmingen), Z-LEHD-FMK (BD Pharmingen), and cyclosporin A (CsA; Cell Signaling Technologies) were dissolved in dimethylsulfoxide (DMSO) and stored at −20°C. The SADS-CoV N protein-specific monoclonal antibody (mAb) was prepared by our laboratory [17]. Antibodies specific for caspase-3, -8 and -9 were obtained from Santa Cruz Biotechnology. The PARP, GAPDH, Fas, FasL, Bid, Bax, Cyt c, apoptosis-inducing factor (AIF), and prohibitin antibodies were purchased from Abcam.

### Transmission electron microscopy (TEM)

Vero E6 cells were pelleted by centrifugation, rinsed thrice with iced phosphate-buffered saline (PBS), fixed with 2.5% glutaraldehyde in 0.1 M phosphate buffer (pH 7.4) overnight, and then postfixed in 2% osmium tetroxide. After dehydration, the samples were embedded in Epon-Araldite. Thin sections were stained with lead citrate and uranyl acetate and then examined with TEM.

### Virus titration

Vero E6 cells were cultured in 96-well plates to 90% confluency and infected with 10-fold serial dilutions of the supernatants. At 4 − 6 days post infection, when the cytopathic effect had stabilized to a constant rate, the cells were analyzed by light microscopy. The TCID_50_/mL was calculated using the Spearman-Kärber method [18].

### DNA fragmentation assay

Low-molecular-weight nuclear DNA was isolated from approximately 10^6^ cells as described by Hinshaw et al. [19], with slight modifications. Briefly, 10^6^ mock-infected or SADS-CoV-infected cells were harvested. The cells were washed in PBS and then resuspended in 500 μL of ice-cold lysis buffer (10 mM Tris [pH 7.5], 1 mM EDTA, 0.2% Triton X-100) containing 500μg/mL protease K for 8−10 h at 55°C. After incubation on ice for 20 min, the lysates were centrifuged at 12,000 *g* at 4°C for 30 min, and the supernatants were extracted with buffered phenol, then with buffered phenol–chloroform, and finally with chloroform-isoamyl alcohol (24:1, vol/vol). DNA was ethanol precipitated with 500 mM NaCl. DNA samples were resuspended in 20 μL of distilled water and treated for 60 min at 37°C with ribonuclease at a final concentration of 20 μg/mL. One-third of the DNA sample was analyzed on a 1.5% agarose gel containing Midori Green Advanced DNA Stain (NIPPON Genetics) in 1 × Tris-borate-EDTA buffer, and the sizes of the oligonucleosomal DNA fragments were estimated using 2-kb markers.

### Terminal deoxynucleotidyl transferase-mediated dUTP-biotin nick end labelling (TUNEL) assay

Apoptotic cells were examined using an In Situ Cell Death Detection Kit, Fluorescein (11684795910; Roche) according to the manufacturer’s instructions. Briefly, Vero E6 or IPI-2I cells were seeded into six-well plates. After infecting with SADS-CoV at an MOI of 0.1, the cells were fixed with 3.7% paraformaldehyde for 60 min at 4°C. After rinsing thrice with PBS, the cells were permeabilized using freshly prepared 0.2% Triton X-100 in 0.1% sodium citrate for 5 min on ice. The cells were then overlaid with 100 μL of TUNEL reaction mixture, according to the manufacturer’s instructions, and incubated for 60 min at 37°C in a humidified atmosphere in the dark. TUNEL-labelled cells were subjected to an immunofluorescence assay using N-specific mAb and Alexa Fluor 594-conjugated goat anti-mouse antibody as described below. Finally, the cells were rinsed five times with PBS and stained with DAPI (4’, 6-diamidino-2-phenylindole) (0.05 μg/mL, Sigma) at room temperature (RT) for 15 min and directly analyzed under a confocal laser Scanning microscope (Zeiss).

### Flow cytometric analysis of apoptosis

Vero E6 or IPI-2I cells were seeded into six-well tissue culture plates for 48 h and mock infected or infected with SADS-CoV at an MOI of 0.1. To examine the effect of each inhibitor on SADS-CoV-induced apoptosis, the cells were treated with Z-VAD-FMK or CsA and then infected with SADS-CoV. The virus-inoculated cells were further propagated in the presence of Z-VAD-FMK, CsA or DMSO. Phosphatidylserine exposure was determined by measuring Annexin V binding at the indicated times using an FITC Annexin V Apoptosis Detection Kit (BD Pharmingen), according to the manufacturer’s manual. Briefly, cells were harvested by centrifugation at 1,500 *g* for 5 min, rinsed once with PBS, and the resuspended in 100 μL of 1 × binding buffer. The cells were then incubated with FITC-conjugated Annexin V and propidium iodide at 25°C for 15 min in the dark. Then, 1 × binding buffer (400 μL) was added to the mixture, and the percentage of apoptotic cells was determined by flow cytometric within 1 h. Cells negative for propidium iodide uptake and positive for Annexin V were considered apoptotic. At least 1 × 10^5^ cells were counted for each data point.

### Experimental infection of piglets and immunohistochemistry (IHC) assay

Eighteen one-day-old specific pathogen-free (SPF) piglets were randomly divided into two groups. The SPF piglets in group 1 were orally inoculated with 5 mL of DMEM, serving as uninfected controls. SPF piglets in group 2 were orally inoculated with 5 mL 10^4^ TCID_50_ SADS-CoV strain. After inoculation, the piglets were observed and recorded thrice daily for clinical symptoms of vomiting, diarrhea, lethargy, and body condition. Three piglets from each group were necropsied at 24, 36 and 48 h post-infection (hpi). All piglets were euthanized according to the Ethical Committee of the Institute. Representative sections of the ileal tissues were fixed with 4% paraformaldehyde and stored in 70% ethanol at 4°C. The IHC assay was performed as previously described [20]. Slides were incubated with mAb 3E9 (1:50) at 4°C overnight and subsequently hybridized with HRP-labelled goat anti-mouse IgG (Sigma) for 1 h. Immunocomplexes were detected using the 3, 3'-diaminobenzidine liquid substrate system.

### Sodium dodecyl sulfate-polyacrylamide gel electrophoresis (SDS-PAGE) and western blot analyses

SDS-PAGE was performed on SDS-12.5% polyacrylamide gels. Proteins separated by SDS-PAGE were electroblotted onto 0.45-μm reinforced nitrocellulose membranes in transfer buffer (0.5 mM Tris-HCl, 0.2 M glycine, 20% methanol) at 300 mA for 120 min using a Mini Trans-Blot Electrophoretic Transfer Cell (Bio-Rad). The membranes were blocked in TBST (20 mM Tris-HCl [pH 7.5], 150 mM NaCl, 0.05% Tween-20) containing 5% nonfat milk at 4°C overnight and then blotted with the primary antibody in TBST buffer for 2 h. After rinsing with TBST five times, the membranes were then transferred to TBST buffer containing 1:10,000-diluted IRDye 800CW goat anti-mouse lgG (H+ L) (1:10,000) (LiCor BioSciences) or IRDye 680RD goat anti-rabbit lgG (H+ L) (1:10,000) (LiCor BioSciences), and thereafter the blots were visualized using an Odyssey infrared imaging system (LiCor BioSciences). Quantification of band intensities by densitometry was performed using the ImageJ software.

### Immunofluorescence assay (IFA)

Vero E6 cells were seeded onto poly-_D_-lysine-coated coverslips at a density of 3 × 10^4^ cells/coverslip. The cells were pretreated with each reagent or DMSO for 1 h and mock infected or infected with SADS-CoV at an MOI of 0.1. The virus-infected cells were subsequently grown in the presence of inhibitors until 36 hpi, fixed with 3.7% paraformaldehyde for 60 min at 4°C and then permeabilized with 1% Triton X-100 in PBS at RT for 5 min. After blocking with 1% goat serum for 30 min, the cells were incubated with mouse anti-SADS-CoV N mAb (1:1000) at 4°C overnight. After rinsing five times in PBS, the cells were incubated for 1 h at RT with a goat anti-mouse secondary antibody conjugated to Alexa Fluor 594 (Invitrogen), followed by counterstaining with DAPI at RT for 15 min. The coverslips were mounted on microscope glass slides in mounting buffer, and cell staining was visualized using an inverted fluorescence microscope (ThermoFisher). For study of colocalization, MitoTracker Red CMXRos (150 nM; Invitrogen) was added to Vero E6 cells under the indicated conditions and left for 60 min at 37°C prior to fixation. The cells were then incubated with rabbit anti-Bax, Cyt c- or AIF-specific antibody, and followed by secondary antibody incubation with goat anti-rabbit antibody conjugated to Alexa Fluor 488 (Invitrogen) as described above. Fluorescence images were captured using a confocal laser scanning microscope (Zeiss). Quantification of the red fluorescence-positive cells was performed by taking the average of at least six fields of view.

### Cell fractionation and subcellular localization of Bax and Cyt c

The cytosolic and mitochondrial fractions were isolated using a Mitochondria/Cytosol Fractionation Kit (BioVision) according to the manufacturer’s manual with some modifications. Briefly, Vero E6 cells were grown in six-well tissue culture plates for 48 h and were mock infected or infected with SADS-CoV at an MOI of 0.1. At the indicated times, the cells were collected by centrifugation at 600 *g* for 10 min at 4°C. The collected cells were washed with ice-cold PBS and then resuspended in 100 μL of lysis buffer containing DTT and protease inhibitors and incubated on ice for 10 min. After complete homogenization, the homogenate was centrifuged at 700 *g* for 10 min at 4°C to remove the nuclei, cellular debris, and intact cells. The supernatant was then collected and centrifuged at 12, 000 *g* for 30 min at 4°C. The supernatant was collected as the cytosolic fraction, and the pellet was resuspended in a mitochondrial extraction buffer mix containing DTT and protease inhibitors (prepared as described in the manufacturer’s instructions) and then stored at −80°C before use. The protein concentration of each fraction was measured using a BCA protein assay (Pierce).

### Cell viability assay

Cell monolayers were incubated with each of four inhibitors (Z-VAD-FMK, Z-IETD-FMK, Z-LEHD-FMK, and CsA) at different concentrations or carrier control DMSO for 36 h. Cell viability was then determined using the Cell Counting Kit-8 (CCK-8) system (CK04; Dojindo) according to the manufacturer’s instructions. Briefly, CCK-8 solution (10 μL per 100 μL of medium in each well) was added, the plates were then incubated at 37°C for 1 h, and the absorbance was read at a wavelength of 450 nm.

### Statistical analysis

Data are shown as the mean ± standard deviation (SD) of three independent experiments done in triplicate. Results were analyzed by one-way ANOVA. Differences with *P* < 0.05 were considered significant.

## Results

### SADS-CoV infection induces apoptosis in Vero E6 and IPI-2I cells (in vitro)

**Figure 1.**
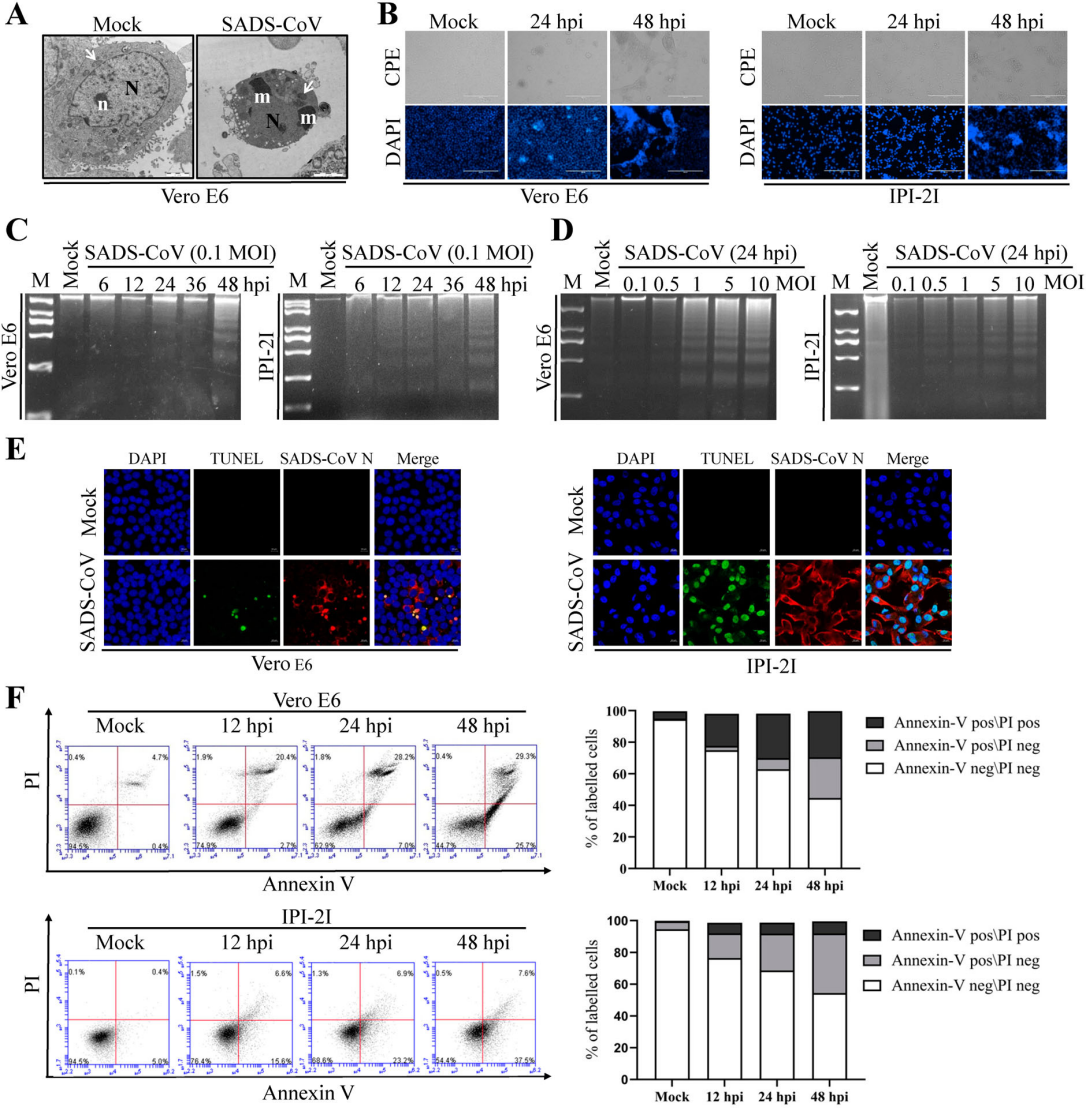
SADS-CoV infection induces apoptosis *in vitro*. (A) Electron micrographs of mock-infected and SADS-CoV-infected Vero E6 cells at 36 hpi. In mock-infected cells (Mock), the round nuclei (N) displays a large, unique, electron-dense nucleolus (n). Mitochondria (arrowheads) are dispersed within the cytoplasm. SADS-CoV-infected cells (SADS-CoV) are characterized by numerous masses of condensed chromatin (m) dispersed at the periphery of a convoluted nucleus (N) and swollen mitochondria (arrowheads) (Bar: 2 μm). (B) Morphological changes in SADS-CoV-infected Vero E6 and IPI-2I cells. Cells were mock or SADS-CoV infected, stained with DAPI at 24 and 48 hpi, and viewed under a light microscope. Phase-contrast images; DAPI, nuclear staining. (C and D) DNA fragmentation in SADS-CoV-infected cells. DNA isolated from SADS-CoV-infected Vero E6 and IPI-2I cells was resolved by 1.5% agarose gel electrophoresis, followed by visualization of bands and photography. Lane M, 2 kb DNA molecular weight marker. Lane mock (Fig.1C), sham-infected for 48 h; lane mock (Fig.1D), sham-infected for 24 h. (E) TUNEL labelling of SADS-CoV-infected cells. Mock-infected control and SADS-CoV-infected cells fixed at 36 hpi were labelled with TUNEL (green) and sequentially stained with an anti-SADS-CoV N antibody (red). Cells were counterstained with DAPI, and photomicrographs of TUNEL labelling and N protein staining in virus-infected cells were obtained using a confocal microscope. (F) Cell death analysis by flow cytometry with dual Annexin V-PI cell labelling. SADS-CoV-infected cells collected at different time points were subjected to dual Annexin V and PI labelling and analyzed by FACS. Lower left quadrants represent intact cells (Annexin V negative\PI negative); Lower right quadrants represent early apoptotic cells (Annexin V positive\PI negative); upper right quadrants indicate late apoptotic and/or necrotic cells (Annexin V positive\PI positive); and upper left quadrants indicate necrotic cells (Annexin V negative\PI positive). The graph on the right represents the percentage of each quadrant, and the non-significant percentages of Annexin V- negative and PI- positive cells were excluded.

To investigate whether SADS-CoV can trigger programmed cell death, virus-infected Vero E6 cells were first examined in terms of ultrastructural alterations. Morphological changes typical of the late stage of apoptosis were only observed with cells fixed at 36 hpi. At that stage, only nucleolar remnants were observed in the convoluted nuclei, which contained small masses of condensed chromatin. The mitochondria were swollen, indicating that these were no longer functional (Figure 1(A)). Furthermore, SADS-CoV-infected cells were examined by optical microscopy after fixation with ethanol and stained with DAPI. Light microscopy of the SADS-CoV-infected Vero E6 and IPI-2I cells indicated that typical CPEs characterized by vacuolation and syncytia formation were visible starting at 24 hpi and became prominent by 48 hpi and most of the infected cells showed a marked nuclear diameter reduction and obvious chromatin condensation 48 h after infection (Figure 1(B)). We then performed two biochemical assays to ascertain whether the morphological changes observed in SADS-CoV infected cells were due to the induction of apoptosis. First, the low-molecular-weight genomic DNA extracted from SADS-CoV-infected Vero E6 and IPI-2I cells at 0.1 MOI for 6, 12, 24, 36, and 48 hpi were analyzed on a 1.5% agarose gel. As shown in Figure 1(C), a canonic oligonucleosome-sized DNA ladder was observed in cells harvested at 48 hpi. In other experiments conducted at MOIs ranging from 0.1 to 10 MOI and at 24 hpi, apoptosis DNA ladders could be detected at MOIs of 1, 5, and 10, and the intensity of the ladder bands increased with dose of infection (Figure 1(D)), confirming that DNA fragmentation was induced by SADS-CoV. No obvious DNA fragmentation was observed in mock-infected cells. Second, nuclear TUNEL staining was performed on mock- and SADS-CoV-infected Vero E6 and IPI-2I cells. As the TUNEL assay could distinguish apoptotic cells undergoing DNA fragmentation by adding labelled nucleotides to the fragmented DNA ends, cells even at the early stages of apoptosis could be visualized by horseradish peroxidase colorimetric reaction. As shown in Figure 1(E), TUNEL signals (DNA damage and dead) were only observed in virus-infected cells compared to mock-infected cells.

To detect the precise apoptotic rate in SADS-CoV-infected Vero E6 and IPI-2I cells, virus- or mock-infected cells were stained with Annexin V and PI and then examined using FACS ﬂow cytometry to quantitatively determine the percentage of viable, apoptotic, and dead cells. Compared with the control, SADS-CoV infection produced a significant level of apoptosis (Annexin V positive\PI negative) at 24 hpi. The percentage of early stage apoptotic cells increased with the progression of infection and reached a maximum of 25.7% and 37.5% in Vero E6 and IPI-2I cells at 48 hpi, respectively (Figure 1(F)). Taken together, these data demonstrate that SADS-CoV infection induced apoptosis *in vitro*.

### SADS-CoV infection activates caspase-8, -9, and -3 and cleaved PARP

**Figure 2.**
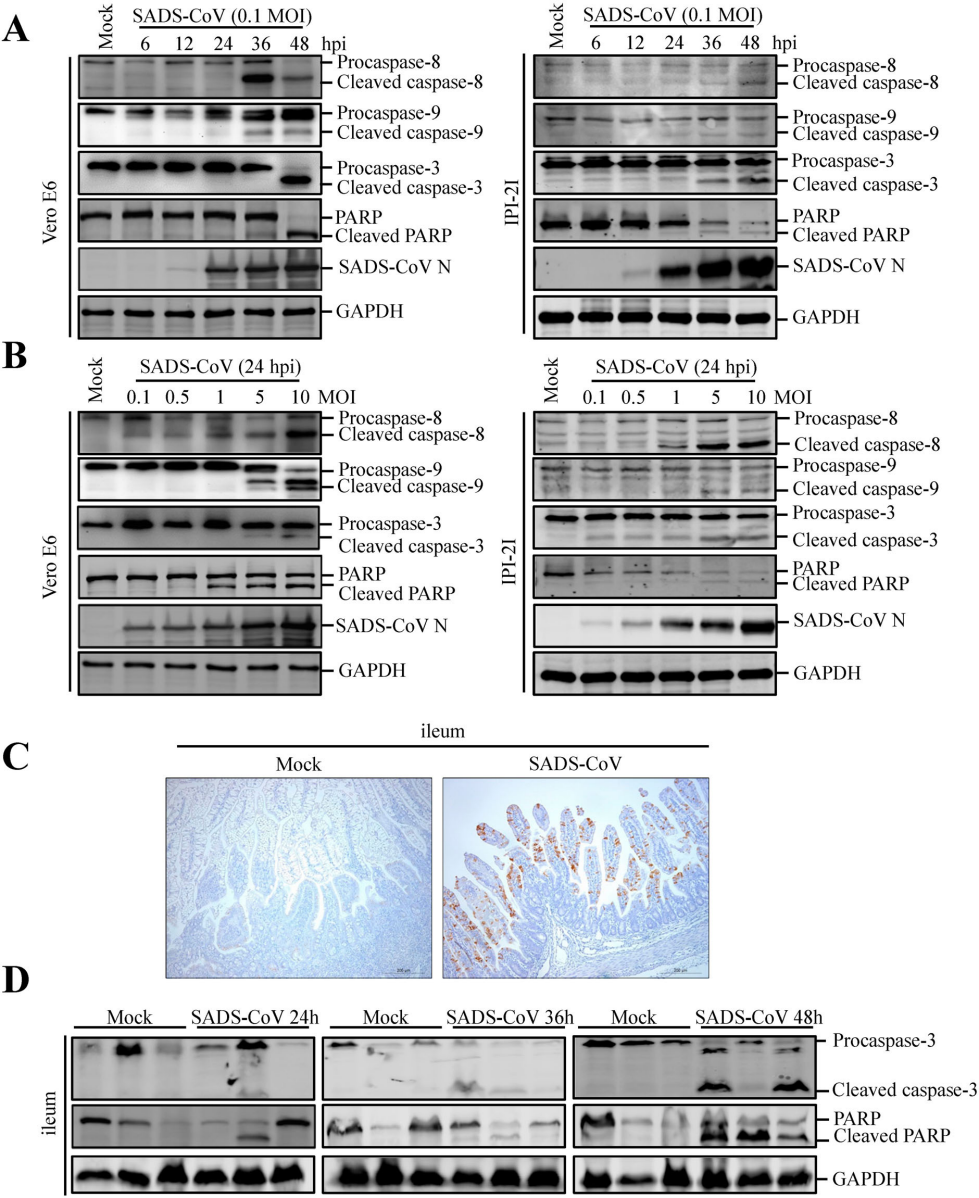
Effects of SADS-CoV infection on caspase activation and PARP cleavage *in vitro* and *in vivo*. (A and B) SADS-CoV infection activates caspase-8, -9, -3 and cleaved PARP *in vitro*. Western blot analysis of caspase activation in SADS-CoV-infected cells (0.1 MOI) at different times or MOIs at 24 hpi. GAPDH was used as internal loading control. (C) Representative microphotographs of viral antigen immunochemical staining in SADS-CoV -non-infected and -infected ileal tissues (Bar: 200 μm). (D) SADS-CoV infection activates caspase-3 and cleaved PARP in vivo. Western blot analysis of the protein expression levels of caspase-3 and cleaved PARP in ileal samples from SADS-CoV -non-infected and -infected piglets at 24, 36, and 48 hpi. GAPDH was used as internal loading control.

Caspases are cysteine proteases that play fundamental roles in the apoptotic responses of cells to different stimuli. To gain insights into the mechanism underlying SADS-CoV-induced apoptosis, we investigated the contribution of caspase to SADS-CoV-induced apoptosis in Vero E6 and IPI-2I cells. Caspase-8, -9 and -3 activity was detected using a panel of caspase-specific antibodies. As shown in Figure 2(A and B), cleaved caspase-8, -9, and -3 was observed in SADS-CoV-infected cells. In addition, their activated form showed a time- and dose-dependent increase. PARP, a representative substrate for effector caspase, can be cleaved by caspase-3 [21]. Western blot analysis showed that the cleaved PARP significantly increased at 48 hpi, whereas no cleaved PARP was detected in mock-infected cells (Figure 2(A)). The abundance of the cleaved fragment of PARP also increased with infection dose in the SADS-CoV-infected cells at 24 hpi (Figure 2(B)).

To explore whether SADS-CoV infection induces apoptosis *in vivo*, apoptosis was monitored in the primary target of SADS-CoV infection *in vivo*, i.e. the ileal tissues of SADS-CoV-infected piglets at 24, 36 and 48 hpi. SADS-CoV infection was confirmed by IHC with a specific mAb to the SADS-CoV N protein (Figure 2(C)). Activated effector caspase-3 and cleaved PARP were verified by western blot in SADS-CoV-infected ileal tissues and their activated form showed a time-dependent increase (Figure 2(D)). Taken together, these results suggest that both extrinsic and intrinsic pathways contribute to caspase-3 activation in SADS-CoV-induced apoptosis *in vitro* and *in vivo*.

### Pan-caspase inhibitor treatment prevents SADS-CoV-induced apoptosis and blocks SADS-CoV infection

**Figure 3.**
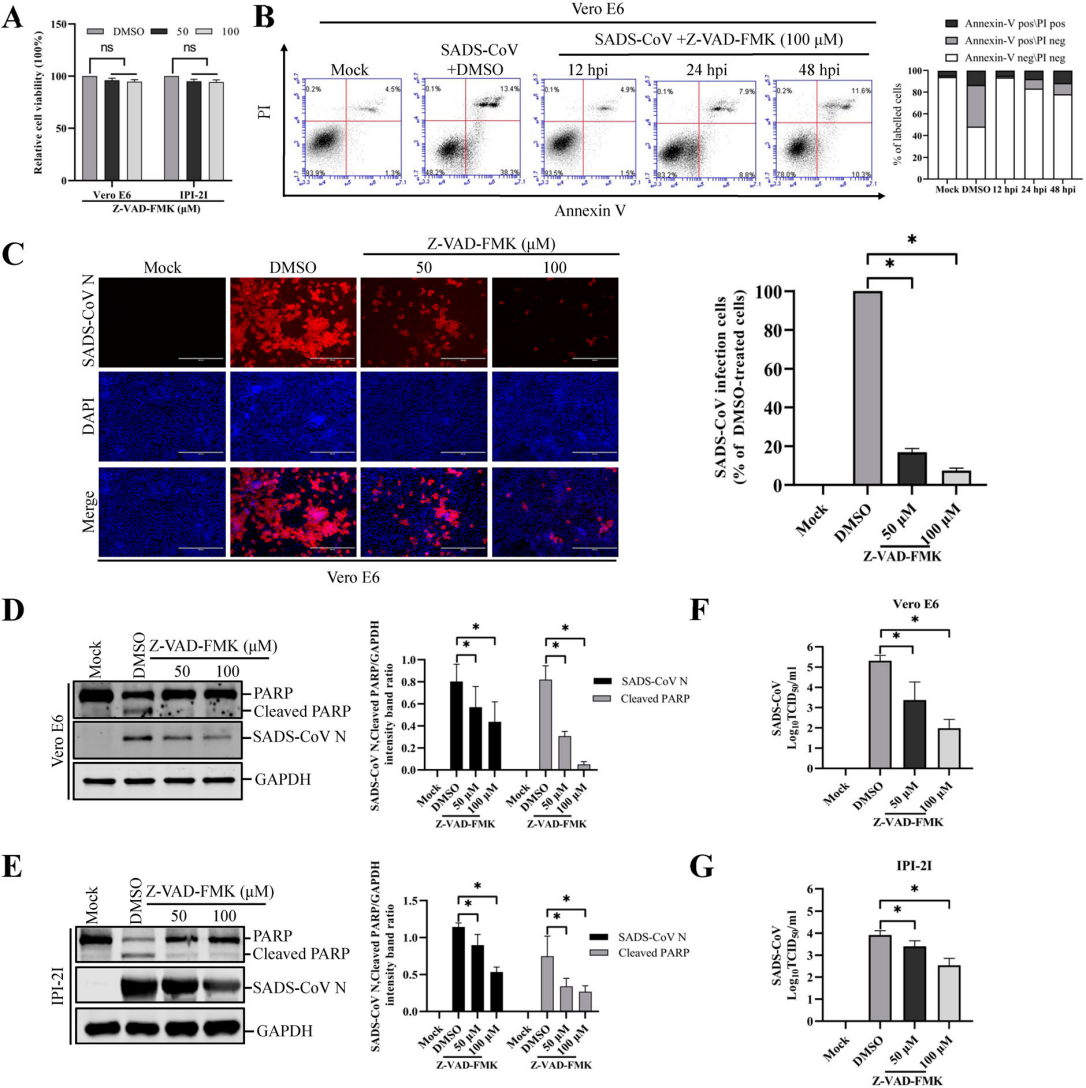
Pan-caspase inhibitor affects SADS-CoV-induced apoptosis and SADS-CoV infection. (A) Z-VAD-FMK treatment does not affect cell viability. Vero E6 and IPI-2I cells were treated with the carrier control DMSO or Z-VAD-FMK at different concentrations for 36 h. Cell cytotoxicity was analyzed by CCK-8 kit as described in Materials and Methods. (B) FACS with dual Annexin V-PI cell labelling in the presence of Z-VAD-FMK. Vero E6 cells were pretreated with DMSO or Z-VAD-FMK (100 μM) for 1 h, followed by mock or SADS-CoV infection. Cells were harvested at the indicated time points, dual labelled with Annexin V and PI, and then analyzed by FACS. The right graph represents the percentage of each quadrant. (C) SADS-CoV replication in the presence of Z-VAD-FMK. Vero E6 cells were treated with DMSO or Z-VAD-FMK at the indicated concentrations for 1 h prior to infection with SADS-CoV. SADS-CoV infected cells were maintained for 36 h in the presence of DMSO or Z-VAD-FMK. For immunostaining, infected cells were fixed at 36 hpi and incubated with mAb against SADS-CoV N protein, followed by incubation with Alexa Fluor 594-conjugated goat anti-mouse secondary antibody. The cells were counterstained with DAPI and examined using an inverted fluorescence microscope. The percentage of SADS-CoV infected cells per view from three independent experiments is expressed as the mean ± SD. (D and E) Viral N protein expression and PARP cleavage in the presence of Z-VAD-FMK. Vero E6 and IPI-2I cells were treated with Z-VAD-FMK at the indicated concentrations for 1 h prior to infection with SADS-CoV. SADS-CoV-infected cells were maintained for 36 h in the presence of DMSO or Z-VAD-FMK. At 36 hpi, cellular lysates were examined by western blot with antibodies against SADS-CoV N protein and PARP. The blot was also reacted with a mouse mAb against GAPDH to verify equal protein loading. Densitometric data of N/GAPDH and cleaved PARP/GAPDH from three independent experiments are expressed as the mean ± SD. (F and G) Z-VAD-FMK treatment suppresses SADS-CoV replication. Treatment and infection conditions were as described for in panel D and E, and the viral titers in the supernatants collected at 36 hpi were determined by the Spearman-Kärber method. Error bars represent the standard errors of the means from three independent experiments.

The central effector machinery of apoptosis is composed of cytoplasmic proteases called caspases. These are present in an inactive form and are irreversibly activated during the effector phase of apoptosis, leading to distinct morphological changes that characterize apoptosis. In humans, 10 caspases have been identified, and some of these can be blocked by specific synthetic peptides [22,23]. To elucidate the mechanism and type of apoptotic cell death, experiments were performed with a broad-spectrum caspase inhibitor, Z-VAD-FMK, which blocks SADS-CoV-induced apoptosis. The inhibitor was used at a concentration of 100 μM, which has been previously shown to completely inhibit protease activity in cultured mammalian cells after induction of apoptosis by various molecules [24–27]. The results of the CCK-8 assay also revealed that none of the tested Z-VAD-FMK doses caused any change in cell viability (Figure 3(A)). Vero E6 cells were treated with 100 μM Z-VAD-FMK and subjected to viral infection. At the indicated time points post-infection, virus-infected and Z-VAD-FMK-treated cells were analyzed by Annexin V/PI flow cytometry for the quantification of Annexin V binding. As shown in Figure 3(B), treatment with Z-VAD-FMK substantially decreased SADS-CoV-induced apoptosis in Vero E6 cells. The percentage of apoptotic cells was significantly reduced in cells treated with Z-VAD-FMK than in the DMSO-treated cells or SADS-CoV-infected cells during the course of infection (compared to Figure 1(F)). These results indicate the association of caspase with SADS-CoV-triggered apoptosis. We next investigated whether the same caspase inhibitor affects SADS-CoV replication. Vero E6 cells were pretreated with Z-VAD-FMK at concentrations of 50 and 100 μM or DMSO for 1 h and then infected with SADS-CoV at an MOI of 0.1. Virus production was confirmed with immunofluorescence and N protein expression at 36 hpi. DMSO-treated control cells were positive for SADS-CoV N protein-specific staining, indicating infection and spread of the virus to neighbouring cells (Figure 3(C)). However, the inhibitory effect of Z-VAD-FMK on viral propagation was clearly detectable. As shown in Figure 3(C), the pan-caspase inhibitor significantly attenuated SADS-CoV N protein expression in a dose-dependent manner. Quantification of N protein staining showed that the proportion (%) of virus-infected cells during markedly decreased ZVAD-FMK treatment. Z-VAD-FMK inhibited viral replication by nearly 90% at the highest concentration tested (Figure 3(C)). The expression level of the SADS-CoV N protein in the presence of Z-VAD-FMK or DMSO was also evaluated by western blot analysis. As shown in Figure 3(D and E), the application of inhibitor Z-VAD-FMK resulted in the downregulation of N protein expression at 36 hpi, accompanied by a significant reduction in viral load in the cell culture supernatants compared with that in the cells treated with DMSO (Figure 3(F and G)). In addition, as revealed in Figure 3(D and E), pretreatment with the inhibitor significantly decreased SADS-CoV-induced cleavage of PARP. Taken together, our data reveal that caspase cascades were activated by SADS-CoV, and their chemical inhibition negatively affected SADS-CoV-induced apoptotic cell death and viral replication, which indicated that caspase activity was required by this process.

### Extrinsic- and intrinsic-dependent apoptosis pathways are required for SADS-CoV infection

**Figure 4.**
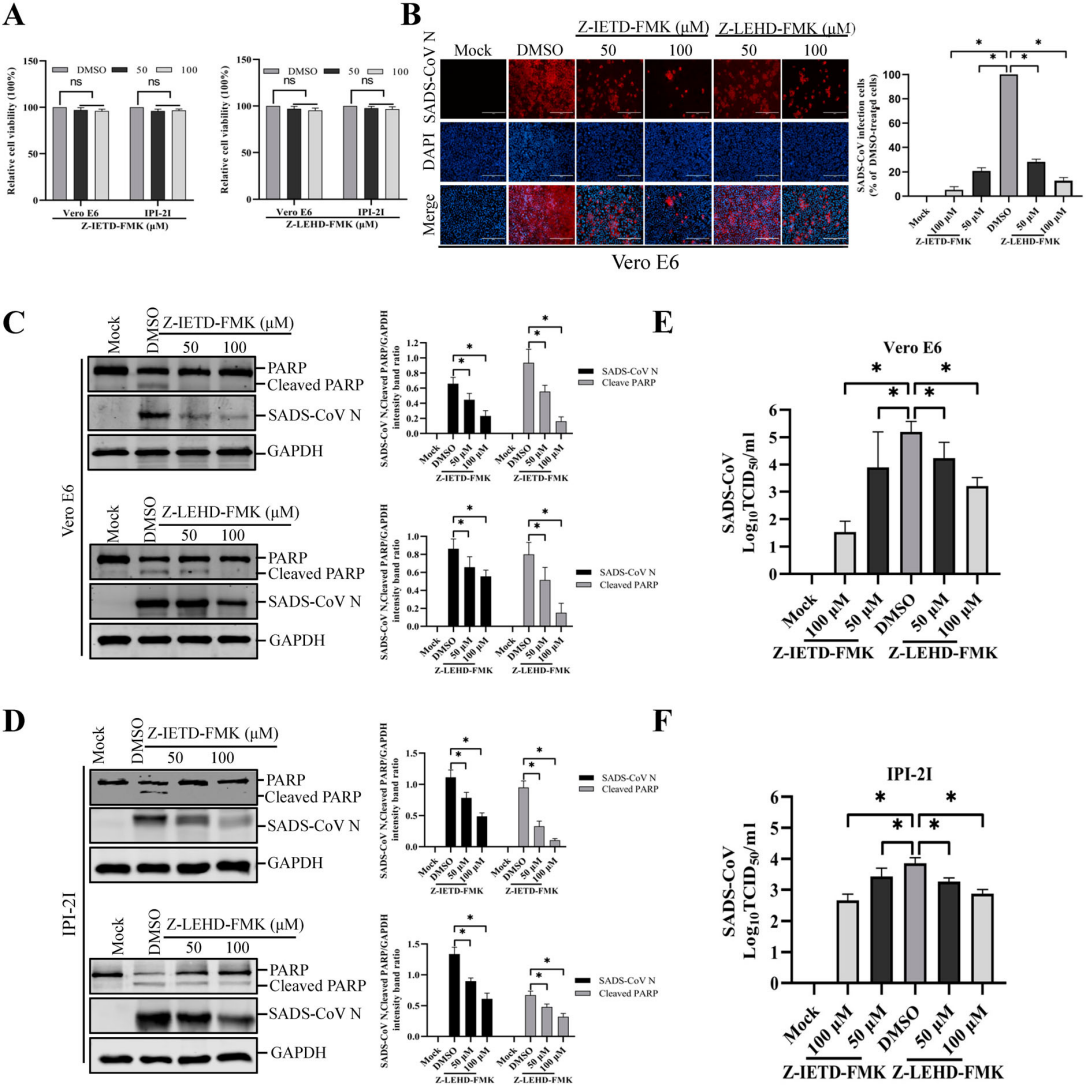
Treatment with caspase-8 and -9 inhibitors attenuates SADS-CoV infection. (A) Z-IETD-FMK and Z-LEHD-FMK treatment does not affect cell viability. Vero E6 and IPI-2I cells were treated with the carrier control DMSO, Z-IETD-FMK or Z-LEHD-FMK at different concentrations for 36 h. Cell cytotoxicity was analyzed by CCK-8 kit as described in Materials and Methods. (B) SADS-CoV propagation in the presence of Z-IETD-FMK (caspase-8 inhibitor) or Z-LEHD-FMK (caspase-9 inhibitor). Vero E6 cells were pretreated with each inhibitor at the indicated concentrations for 1 h and then infected with SADS-CoV. SADS-CoV -infected cells were further maintained for 36 h in the presence of DMSO, Z-IETD-FMK, or Z-LEHD-FMK. At 36 hpi, virus-infected cells were subjected to IFA with an anti-SADS-CoV N antibody, followed by DAPI counterstaining and examination under an inverted fluorescence microscope. The percentage of SADS-CoV infected cells per view from three independent experiments is expressed as the mean ± SD. (C and D) Viral N protein expression and PARP cleavage in the presence of Z-IETD-FMK or Z-LEHD-FMK. Vero E6 and IPI-2I cells were treated with DMSO, Z-IETD-FMK or Z-LEHD-FMK at the indicated concentrations for 1 h prior to infection with SADS-CoV. SADS-CoV infected cells were maintained for 36 h in the presence of DMSO, Z-IETD-FMK or Z-LEHD-FMK. At 36 hpi, cellular lysates were examined by western blot with antibodies against SADS-CoV N protein and PARP. The blot was also reacted with a mouse mAb against GAPDH to verify equal protein loading. Densitometric data for N/GAPDH and cleaved PARP/GAPDH from three independent experiments are expressed as the mean ± SD. (E and F) Z-IETD-FMK or Z-LEHD-FMK treatment suppresses SADS-CoV replication. Treatment and infection conditions were as described for in panel C and D, and the viral titers in the supernatants collected at 36 hpi were determined by the Spearman-Kärber method. Error bars represent the standard errors of the means from three independent experiments.

Apoptosis can be triggered by two distinct signaling pathways, namely, the extrinsic and intrinsic pathways. Although both pathways engage the same proteolytic caspase cascade and interface at the point of downstream executioner caspase-3 activation, initiator caspase-8 and caspase-9 discriminate between the extrinsic and intrinsic pathways [28]. To identify the apoptosis pathway induced by SADS-CoV, specific inhibitors of caspase-8 or caspase-9 were used. The cytotoxic effect of Z-IETD-FMK and Z-LEHD-FMK on Vero E6 and IPI-2I cells was examined by CCK-8. These two inhibitors did not impart significant toxicity on the two cell lines at concentrations of 50 and 100 μM (Figure 4(A)). Vero E6 cells were pretreated with Z-IETD-FMK (caspase-8 inhibitor) or Z-LEHD-FMK (caspase-9 inhibitor) at concentrations of 50 and 100 μM or DMSO, followed by viral infection. The results in Figure 4(B) show that Z-IETD-FMK effectively suppressed SADS-CoV N protein expression, whereas Z-LEHD-FMK imparted a weak effect on N protein expression. The expression level of the SADS-CoV N protein in the presence of Z-IETD-FMK, Z-LEHD-FMK, or DMSO was also evaluated by western blot in Vero E6 and IPI-2I cells. As shown in Figure 4(C and D), inhibitor Z-IETD-FMK or Z-LEHD-FMK at the highest concentrations resulted in the downregulation of N protein expression at 36 hpi. Virus yield was also determined during treatment with initiator caspase inhibitors. After infection, viral supernatants were collected at 36 hpi and viral titers were measured. As illustrated in Figure 4(E and F), caspase-8 and -9 inhibitors suppressed the release of viral progeny in a dose-dependent manner. Meanwhile, when cells infected with SADS-CoV were treated with a caspase inhibitor at concentrations of 50 and 100 μM, both caspase inhibitors significantly decreased SADS-CoV-induced PARP cleavage (Figure 4(C and D)). These findings indicate that, in Vero E6 and IPI-2I cells, SADS-CoV may induce apoptosis through both caspase-8 and caspase-9 dependent pathways. These findings indicate that the death receptor-mediated extrinsic pathway and mitochondrial-mediated intrinsic pathway are both activated, and there is significant crosstalk between the two pathways, which may mean that tBid transmits signals from the extrinsic (caspase-8) to the intrinsic pathway (caspase-9) in SADS-CoV-induced apoptosis [29].

### SADS-CoV infection induces apoptosis via the Fas/FasL-dependent pathway and Bid cleavage

**Figure 5.**
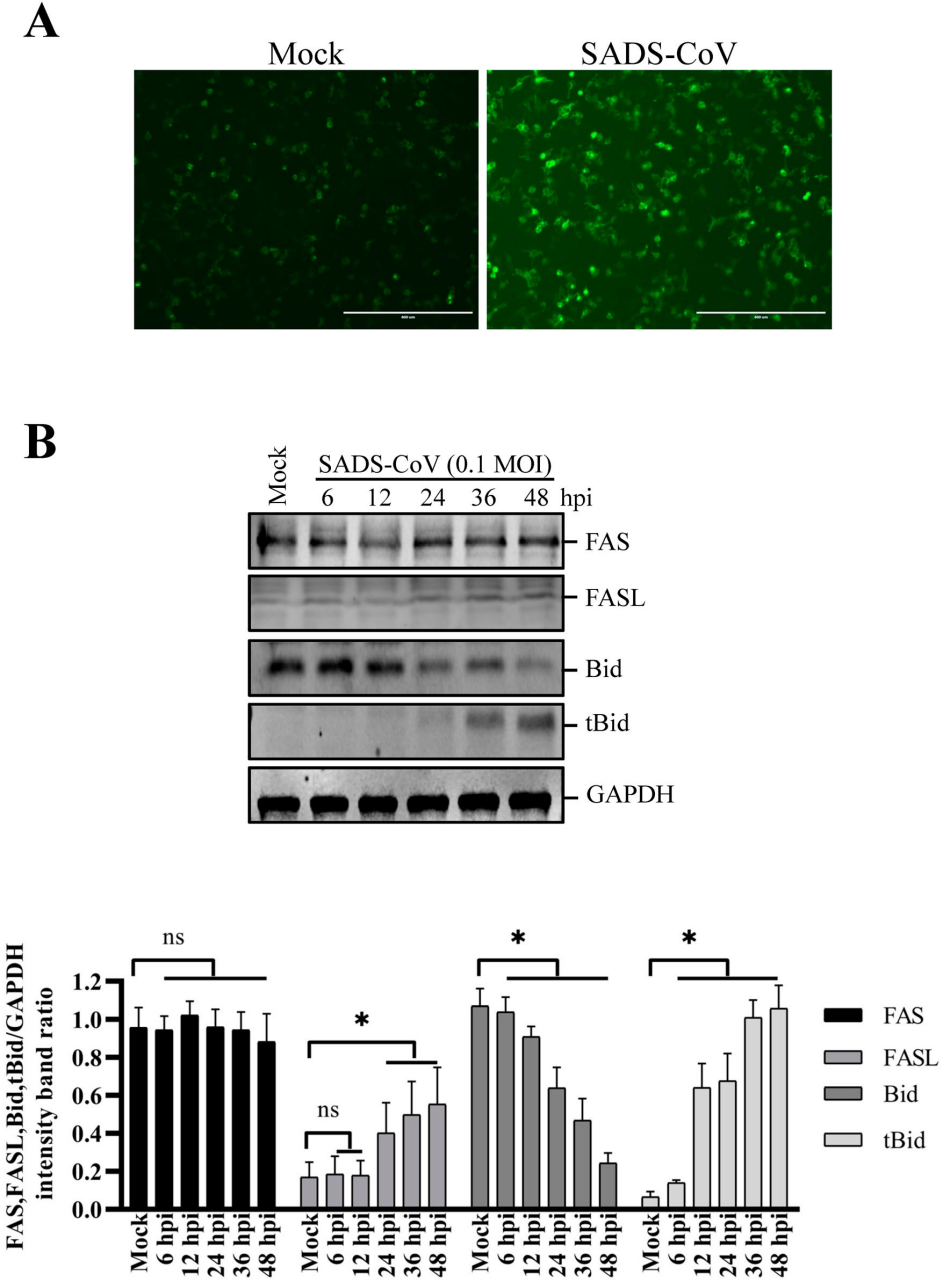
Effects of SADS-CoV infection on Fas, FasL expression and Bid cleavage in Vero E6 cells. (A) SADS-CoV increases cell surface expression of FasL. Cells were fixed and stained for FasL at 36 hpi and observed under fluorescence microscopy. (B) Cells were mock or SADS-CoV-infected at 0.1 MOI at different time points. Cell lysates were analyzed by western blot. Densitometric data for Fas, FasL, Bid, and tBid/GAPDH from three independent experiments are expressed as the mean ± SD.

The results presented here demonstrate that SADS-CoV infection activates both caspase-8- and caspase-9-mediated apoptosis pathways. Caspase-8 plays a pivotal role in Fas/FasL mediated-apoptosis. The involvement of caspase-8 prompted us to further test whether Fas and FasL are involved in SADS-CoV-induced apoptosis. For that purpose, cell surface expression of Fas and FasL was determined by IFA and western blot. As shown in Figure 5(A), enhanced expression of FasL was evident on the cell surface of SADS-CoV-infected cells at 36 hpi. In this experiment, cells were not co-stained with anti-SADS-CoV N antibody because antibodies available for anti-SADS-CoV N protein staining require permeabilization for intracellular staining. At 36 hpi, about 90% of the cells were positively stained for the nucleocapsid protein of SADS-CoV (Figure 3(C)). Therefore, we concluded that most of cells with upregulated FasL are SADS-CoV-infected cells. Furthermore, the expression level of Fas and FasL in the SADS-CoV-infected Vero E6 cells was also evaluated by western blot analysis. As shown in Figure 5(B), the protein levels of FasL significantly increased in a time-dependent manner, whereas no changes in Fas expression were observed after SADS-CoV infection, suggesting that FasL expression is upregulated in the SADS-CoV-infected cells. In subsequent experiments, we investigated whether there was crosstalk between the extrinsic- and intrinsic-dependent apoptosis pathways. It has been shown that the death receptor-mediated activation of caspase-8 cleaves Bid, which is a Bcl-2 family member. Cleaved Bid that is translocated to mitochondria participates in the destruction of mitochondria integrity and Cyt c release to cytosol, which in turn facilitate caspase-9 activation [30]. Therefore, to test this possibility in SADS-CoV-induced apoptosis, we investigated whether Bid was cleaved upon SADS-CoV infection. Western blot analysis revealed that tBid was not detected in uninfected cells but that SADS-CoV infection resulted in cleavage of the full-length 22 kDa Bid to 15 kDa tBid at 24, 36, and 48 hpi, possibly by activated caspase-8 (Figure 5(B)). Taken together, these results imply that apoptosis signals from FasL are transmitted to activate caspase-8, which in turn cleaves Bid. Cleaved Bid activates caspase-9, which links the extrinsic and intrinsic pathways.

### SADS-CoV infection promotes Bax and Cyt c relocalization in Vero E6 cells, but not AIF

**Figure 6.**
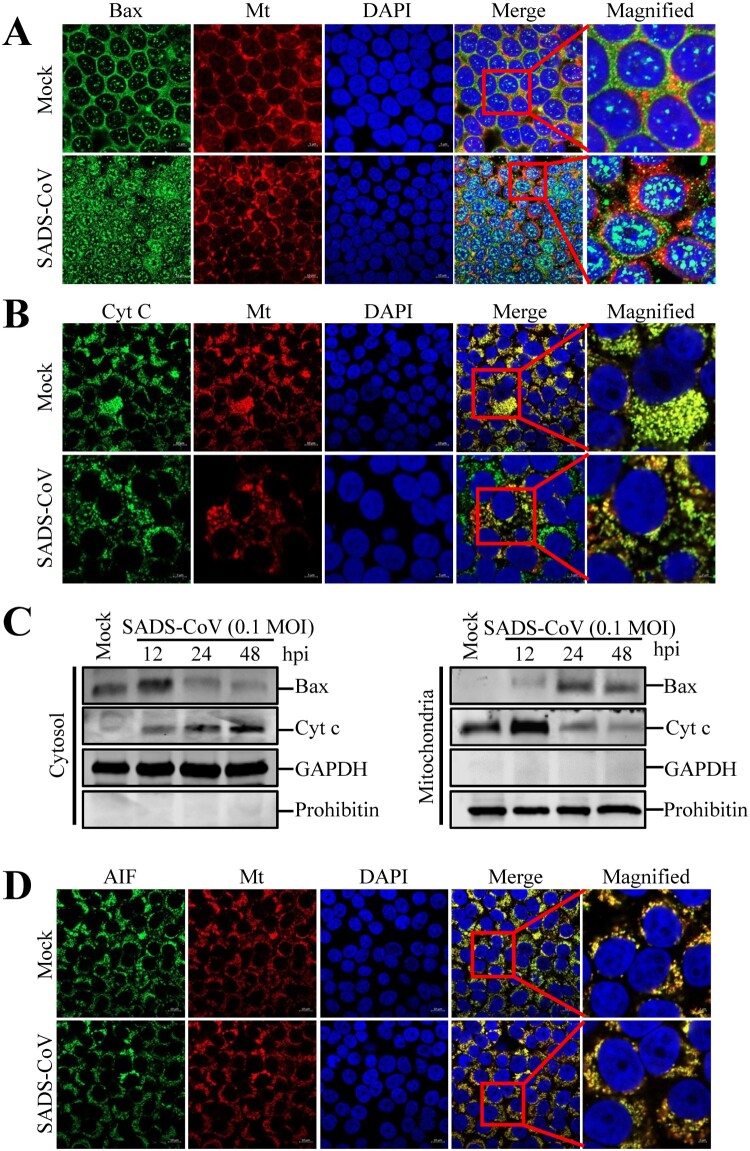
Infections by SADS-CoV promote Bax and Cyt c relocalization but not AIF relocalization in Vero E6 cells. (A and B) Immunofluorescent detection of activated Bax and Cyt c. At 36 hpi, mock-infected or SADS-CoV-infected Vero E6 cells were incubated with the MitoTracker Red CMXROS (red), fixed, and incubated with anti-activated Bax or Cyt c antibodies (green). Bax mitochondrial relocalization is represented as the merger of Bax and mitochondrial marker (yellow), while the residual cytosolic localization is indicated by single staining signal (green). Conversely, Cyt c cytosolic relocalization is represented by single staining signals (green), and residual mitochondrial accumulation is indicated as the colocation of Cyt c and MitoTracker Red CMXROS (yellow). The square-enclosed region provides a higher-magnification view. (C) Western blot analysis of Bax and Cyt c. The mitochondrial and cytosolic fractions were subjected to western blot with an antibody specific to Bax, Cyt c, GAPDH (cytosolic protein marker), or prohibitin (mitochondrial protein marker). All subcellular protein markers served as loading controls. (D) Immunofluorescent detection of activated AIF. At 36 hpi, mock or SADS-CoV-infected Vero E6 cells were incubated with MitoTracker Red CMXROS (red), fixed, and incubated with anti-AIF antibody (green). AIF mitochondrial retention is represented as the merger of AIF and mitochondrial marker (yellow). The square-enclosed region provides a higher-magnification view.

The mitochondrion is thought to play a central role in apoptosis by releasing proapoptotic factors, such as Cyt c and AIF, after organelle permeabilization [31]. Given that Bax is one of the major proapoptotic factors promoting permeabilization of the mitochondrial outer membrane [32], we analyzed its translocation to the mitochondria. Using antibodies specific to the activated form of Bax and Cyt c, immunofluorescence confocal microscopy revealed that Bax continued to be largely localized in the mitochondria after SADS-CoV infection, as indicated by its colocalization with a mitochondrial-specific marker. However, Cyt c was present in the cytosolic region and failed to colocalize with the mitochondria marker in cells infected with SADS-CoV (Figure 6(A and B)). These results were confirmed by western blot analysis of mitochondrial and cytosolic extracts, which indicated that SADS-CoV-infected cells exhibited an independent increase in the expression levels of Bax and Cyt c in the mitochondrial and cytosolic extracts (Figure 6(C)). To eliminate the possibility of association between caspase-independent processes and SADS-CoV-triggered apoptosis, we analyzed the translocation of another proapoptotic protein, namely, AIF, which is proteolytically cleaved and released into the nucleus by mitochondrial outer membrane permeabilization (MOMP) to execute caspase-independent intrinsic apoptosis [1]. Using immunofluorescence confocal microscopy, we demonstrated that AIF was detected in the mitochondria but absent in the nucleus throughout the SADS-CoV infection period, as demonstrated from AIF and MitoTracker colocalization (Figure 6(D)). AIF retention in the mitochondria during SADS-CoV infection was coincided with the execution of caspase-dependent apoptotic cell death by SADS-CoV as earlier described. Thus, SADS-CoV infection triggers Bax recruitment into the mitochondria, leading to MOMP and the consequent release of mitochondrial Cyt c to commit cells to death.

### Mitochondrial permeability transition pore (MPTP) inhibition blocks SADS-CoV-induced apoptosis and SADS-CoV replication

**Figure 7.**
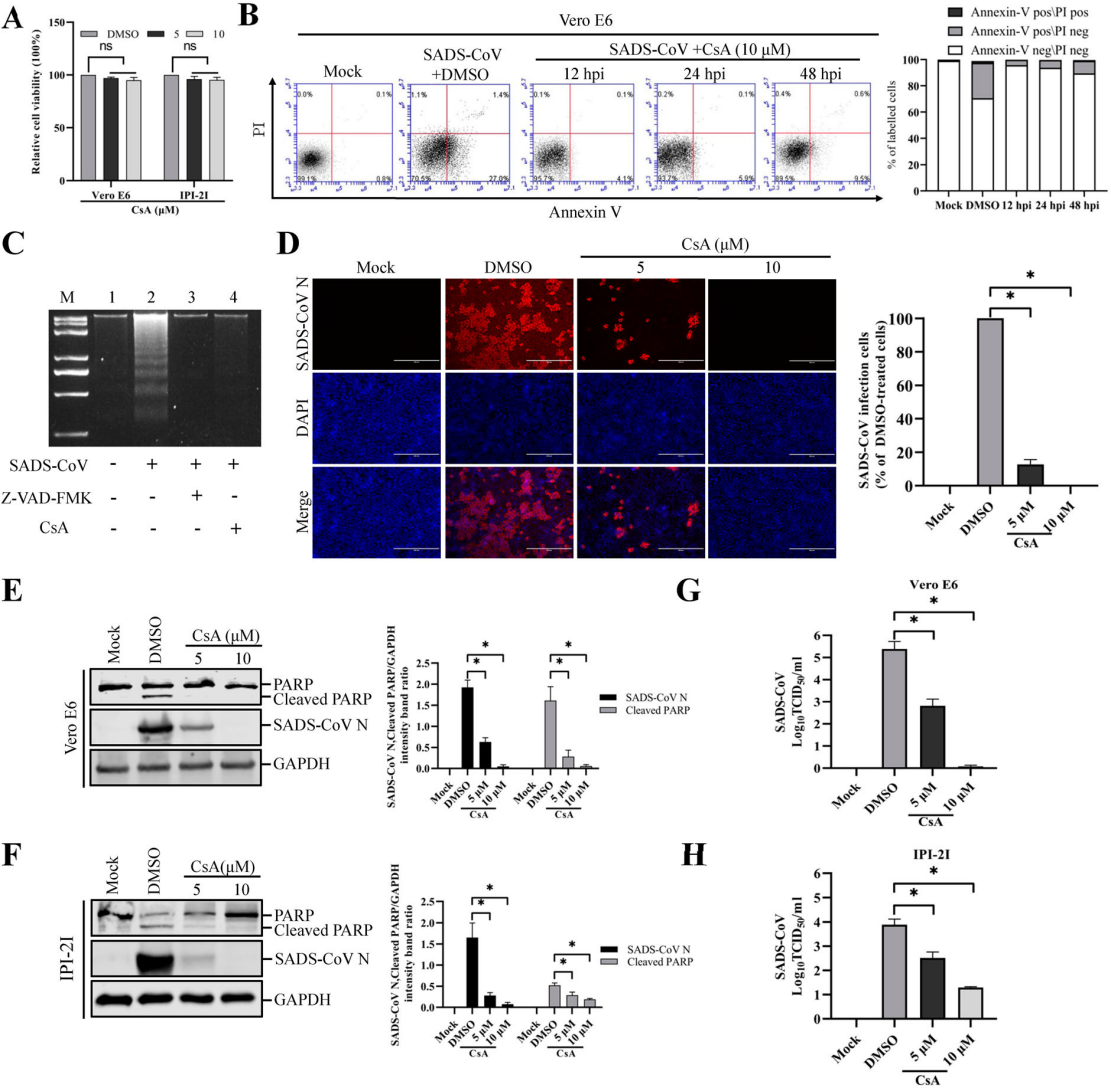
CsA treatment diminishes SADS-CoV-induced apoptosis and suppresses SADS-CoV propagation. (A) CsA treatment does not affect cell viability. Vero E6 and IPI-2I cells were treated with the carrier control DMSO or CsA at different concentrations for 36 h. Cell cytotoxicity was analyzed by CCK-8 kit as described in Materials and Methods. (B) FACS with dual Annexin V-PI cell labelling in the presence of CsA. Vero E6 cells were pretreated with DMSO or CsA (10 μM) for 1 h and mock-infected or infected with SADS-CoV in the presence of DMSO or CsA. Cells were harvested at the indicated time points, dual labelled with Annexin V and PI, and analyzed by FACS. The graph on the right represents the percentage of each quadrant. (C) DNA fragmentation analysis in the presence of CsA. Vero E6 cells were pre-incubated with Z-VAD-FMK (100 μM) or CsA (10 μM) for 1 h and infected with mock virus or SADS-CoV. Nucleosomal DNA fragmentation of the cells was analyzed by agarose gel electrophoresis. Lane M, 2-kb DNA molecular weight marker; lane 1, mock-infected and non-treated; lane 2, only SADS-CoV-infected; lane 3, SADS-CoV-infected and Z-VAD-FMK-treated; lane 4, SADS-CoV-infected and CsA-treated. (D) SADS-CoV infection in the presence of CsA. Vero E6 cells were treated with DMSO or CsA at the indicated concentrations for 1 h prior to their infection with SADS-CoV. SADS-CoV-infected cells were further maintained for 36 h in the presence of DMSO or CsA. For immunostaining, infected cells were fixed at 36 hpi and stained with an anti- SADS-CoV N protein antibody, followed by incubation with Alexa Fluor 594-conjugated goat anti-mouse secondary antibody. The cells were counterstained with DAPI and examined under an inverted fluorescence microscope. The percentage of SADS-CoV infected cells per view from three independent experiments is expressed as the mean ± SD. (E and F) Viral N protein expression and PARP cleavage in the presence of CsA. Vero E6 and IPI-2I cells were treated with DMSO or CsA at the indicated concentrations for 1 h prior to infection with SADS-CoV. SADS-CoV infected cells were maintained for 36 h in the presence of DMSO or CsA. At 36 hpi, cellular lysates were examined by western blot with antibodies against SADS-CoV N protein and PARP. The blot was also reacted with a mouse mAb against GAPDH to verify equal protein loading. Densitometric data for N/GAPDH and cleaved PARP/GAPDH from three independent experiments are expressed as the mean ± SD. (G and H) CsA treatment suppresses SADS-CoV replication. Treatment and infection conditions were as described in panel E and F, and the viral titers in the supernatants collected at 36 hpi were determined by the Spearman-Kärber method. Error bars represent the standard errors of the means from three independent experiments.

The present study demonstrated the translocation of mitochondrial Cyt c into the cytoplasm by Bax-mediated MOMP following SADS-CoV infection. Along with this mechanism, the MPTP is considered as the most viable model of mitochondrial channels that accounts for MOMP [33]. Cyclophilin D (CypD) is located in the mitochondrial membrane matrix and is the main component in the process of MPTP opening [34]. Therefore, we evaluated whether CypD inhibition may prevent SADS-CoV-induced apoptotic cell death. Vero E6 and IPI-2I cells were treated with cyclosporine A (CsA), a chemical inhibitor of CypD, to prevent MPTP opening upon SADS-CoV infection. The cytotoxic effect of CsA on Vero E6 and IPI-2I cells was also examined using CCK-8. This inhibitor showed no significant toxicity on the two cell lines at concentrations of 5 and 10 μM (Figure 7(A)). Following CsA treatment, SADS-CoV-induced apoptosis was quantitatively measured by Annexin V/PI flow cytometry. As shown in Figure 7(B), treatment with CsA notably decreased the percentage of SADS-CoV-induced apoptotic cells during the course of SADS-CoV infection. DNA laddering assay indicated that Z-VAD-FMK or CsA treatment in SADS-CoV infected cells completely abolished intracellular DNA fragmentation (Figure 7(C), lanes 3 and 4), whereas cells infected with SADS-CoV alone clearly displayed a DNA laddering pattern (Figure 7(C), lane 2). These results demonstrate that CsA-mediated CypD inhibition efficiently suppressed apoptotic cell death induced by SADS-CoV, suggesting that SADS-CoV infection triggers the mitochondrial apoptotic pathway.

To examine the effect of CsA on SADS-CoV replication, cells were pretreated with CsA at concentrations of 5 and 10 μM, and CsA was present during the entire period of infection. SADS-CoV replication was verified by immunofluorescence at 36 hpi (Figure 7(D)). In contrast to DMSO-treated control cells, CsA had a strong inhibitory effect on SADS-CoV proliferation by significantly reducing N protein expression. The number of cells expressing viral antigen, as quantified by N protein staining, decreased during CsA treatment, with almost 100% inhibition observed at the 10 μM concentration (Figure 7(D)). To further assess the antiviral activity of CsA against SADS-CoV replication, virus N protein expression was determined in the presence or absence of CsA in Vero E6 and IPI-2I cells. Western blot analysis revealed that 10 μM CsA treatment significantly prevented the intracellular expression of the viral N protein (Figure 7(E and F)). In addition, as revealed in Figure 7(E and F), pretreatment with the inhibitor significantly decreased SADS-CoV-induced cleavage of PARP. After infection, the viral supernatants were collected at 36 hpi and viral titers were measured. As shown in Figure 7(G and H), CsA treatment significantly reduced the release of viral progeny in a dose-dependent manner. Taken together, CsA proficiently suppressed SADS-CoV replication and inhibited SADS-CoV-induced apoptosis.

## Discussion

Apoptosis is a tightly controlled multistep process of cell death that occurs in response to a wide range of stimuli, including viral infections. Viruses possess various mechanisms to inhibit apoptosis, thereby allowing them to evade the innate immune defenses, which restrict viral infection by eliminating infected cells through the interactions at different stages of the apoptosis pathway. However, some viruses induce apoptosis to facilitate the release and dissemination of viral progeny for further invasion, which are important biological activities in viral pathogenesis and disease processes that promote cell death and tissue injury. Recent studies have demonstrated the ability of numerous coronaviruses to elicit or inhibit apoptosis either directly or indirectly during their replication cycles such as transmissible gastroenteritis virus (TGEV) [10,35], avian infectious bronchitis virus (IBV) [36], mouse hepatitis virus (MHV) [37], porcine deltacoronavirus (PDCoV) [12], and porcine epidemic diarrhea virus (PEDV) [11]. Although various viruses are known to modulate apoptosis as a critical armament to complete their replication cycle, whether SADS-CoV induces apoptosis remains unclear. Current available information on SADS-CoV is limited, yet this virus has been associated with severe and acute diarrhea and acute vomiting [15]. Moreover, in contrast to human diseases for which a considerable number of studies have been conducted on the relationship between apoptosis and viral diseases, the relationship between apoptosis and pig infections remains largely unknown.

Using Vero E6 and IPI-2I cells, which are permissive to SADS-CoV, we characterized apoptotic death of infected cells. Morphologic and biochemical features of apoptosis such as blebbing of the plasma membrane, translocation of phosphatidylserine to cell surface and Annexin V staining, nuclear fragmentation, apoptotic body formation, and DNA laddering were assessed in SADS-CoV-infected cells. The findings of this study strongly suggest that the cytopathology of SADS-CoV infection, which is represented by vacuolation and syncytia formation *in vitro*, is associated with apoptosis.

The morphological characteristics of apoptosis are typically caused by the sequential activation of caspases, which are normally present in mammalian cells as inactive precursors [28]. Caspases, a family of cysteine-dependent aspartate-directed proteases, play pivotal roles in the initiation and execution of apoptosis by cleaving a large number of proteins [21]. The activation of the caspase cascades is a trigger for apoptosis in some viruses-infected cells [6,38,39]. SADS-CoV infection activated caspase-8, -9 and -3 and cleavage of a major caspase substrate, PARP *in vitro* and *in vivo*, which provides additional evidence for caspase activation. These results suggest that the activation of caspase cascades is involved in SADS-CoV-induced apoptosis *in vitro* and *in vivo*. Similar results have been found in other coronaviruses, including canine coronavirus, IBV, and TGEV [10,36,39].

To investigate the contribution of caspases to SADS-CoV-induced apoptosis, cells infected with SADS-CoV were treated with a broad spectrum caspase inhibitor. This caspase inhibitor significantly attenuated the apoptotic effects of SADS-CoV infection. These results suggest that the activation of caspase cascades is involved in SADS-CoV-induced apoptosis in Vero E6 and IPI-2I cells. Both extrinsic and intrinsic apoptotic pathway may activate the corresponding upstream initiator caspases, leading to the processing and activation of downstream common effector caspases to execute apoptosis [1,28]. Numerous studies have demonstrated that viruses also use either intrinsic or extrinsic pathways, or both, to induce apoptosis. Viruses such as VSV, rhinovirus, and parvovirus B19 induce apoptosis via the caspase-9 mediated intrinsic pathway [40–42]. Extrinsic pathway activity via caspase-8 activation mediates apoptosis in HIV, lyssavirus, Tula hantavirus, Sendai virus, and influenza virus [43–47]. Treatment of cells with caspase-8 and -9 inhibitors effectively restricted SADS-CoV infection, which is indicative of the involvement of the extrinsic and intrinsic apoptotic pathway in SADS-CoV infection. Virus-induced apoptosis is an important and complex aspect of the pathogenesis of viral infections. There is a long-standing mystery regarding the linkage between apoptosis and virus replication. Although many viruses have developed mechanisms to suppress apoptosis for their own benefit [48], evidence has emerged for viral hijacking of host caspases for facilitating viral replication [49]. Therefore, the role of apoptosis in SADS-CoV progeny release was studied here. When PARP cleavage in SADS-CoV-infected cells was blocked, SADS-CoV infection was dramatically inhibited. These findings suggested that apoptosis is required for the replication of SADS-CoV as shown in PDCoV [12]. Thus, the caspase-dependent apoptotic pathway seems to be manipulated by SADS-CoV to ensure competent viral replication in target cells.

Fas/FasL-mediated signaling has been reported in apoptosis in response to infection of several viruses such as TGEV [10], murine coronavirus [50], and bovine ephemeral fever virus [51]. Caspase-8 activation by SADS-CoV implicated the involvement of a specific death receptor. Following SADS-CoV infection, FasL expression was notably increased, whereas Fas expression slightly changed. This finding indicates that caspase-8 activation in SADS-CoV-infected cells could be mediated by the ligation of Fas and FasL. Future studies will determine the role of FasL in SADS-CoV-induced apoptosis using decoy receptors and neutralizing antibodies. In most cases of virus-induced apoptosis, apoptosis is a result of crosstalk between intrinsic and extrinsic pathways [6,39]. In this process, activated caspase-8 cleaves Bid to tBid, which is then translocated to the mitochondria and initiates the release of Cyt c into the cytosol, leading to caspase-9 activation [30]. The potential crosstalk between intrinsic and extrinsic pathways was proven by the cleavage of Bid in SADS-CoV-infected cells. Similar results have been demonstrated for hepatitis C virus and BVDV [52,53].

In response to various intracellular apoptotic signals, mitochondria undergo loss of inner mitochondrial membrane potential and subsequently release several pro-apoptotic proteins [54]. Among these, cytosolic Cyt c participates with other pro-apoptotic factors in the formation of the apoptosome, which triggers the caspase-dependent proteolytic cascade [55], whereas AIF critically functions in caspase-independent apoptosis by relocating to the nucleus [1]. Evidence for the induction of caspase-dependent and –independent mitochondrial pathways by viral infection has been reported, such as human immunodeficiency virus and VSV [41,56]. However, the translocation of AIF during viral infection does not generally occur. For example, herpes simplex virus or bovine ephemeral fever virus-mediated apoptosis does not require AIF translocation [51,57]. Recently, porcine epidemic diarrhea virus has been reported to induce apoptosis associated with translocation of AIF, but not Cyt c [11]. As caspases are relevant to both SADS-CoV-induced apoptosis and viral replication, we investigated the status of Cyt c release from the mitochondria of SADS-CoV-infected cells. The translocation of Bax into the mitochondria was observed during the early stage of SADS-CoV infection, wherein it likely participates in the formation of pores in the mitochondrial outer membrane. At the same time, the majority of Cyt c was detected and retained thereafter in the cytoplasm of SADS-CoV-infected cells. In contrast, the displacement of AIF from the mitochondria to the nucleus was completely absent in SADS-CoV-infected cells. This result further supports the hypothesis that SADS-CoV induced apoptosis involves caspase-dependent mitochondrial apoptotic pathways.

Apoptosis is controlled at the mitochondrial level by the sequestration of apoptogenic proteins (such as Cyt c) in the mitochondrial intermembrane space, and the cytosolic release of these factors upon exposure to proapoptotic signals [58,59]. The release of Cyt c is ultimately controlled by the highly conserved Bcl-2 family of proteins, which regulated the integrity of the mitochondria [60,61]. Some viruses are found to induce apoptosis through Bax and/or Bak activation, such as hepatitis C virus, West Nile virus and VSV [62–64]. Bax plays a key regulatory role in inducing MOMP, a process distinct from the MPTP [65]. The relocation of Cyt c leads us to hypothesize that MOMP is regulated by the opening process following MPTP formation during SADS-CoV infection, in which CypD functions as a major inducing factor to constitute the MPTP complex in the mitochondrial membrane. CsA acts as a specific inhibitor of MPTP opening by inhibiting the activity of CypD, which constitutes the MPTP complex in the mitochondrial inner membrane. Our results show that the pharmacological inhibition of MPTP opening with CsA suppressed SADS-CoV-induced apoptosis. CsA is known to inhibit the replication of several RNA viruses, including nidoviruses, by affecting the function of various members of the cellular cyclophilin protein family [66–70]. We showed here that CsA exerted its antiviral activity through the inhibition of multiple steps of the SADS-CoV life cycle, including viral protein translation and the spread of viral progeny. Therefore, CsA appears to synergistically elicit its antiviral activity on the replication of SADS-CoV through the inhibition of apoptotic cell death and interference of viral protein synthesis.

**Figure 8.**
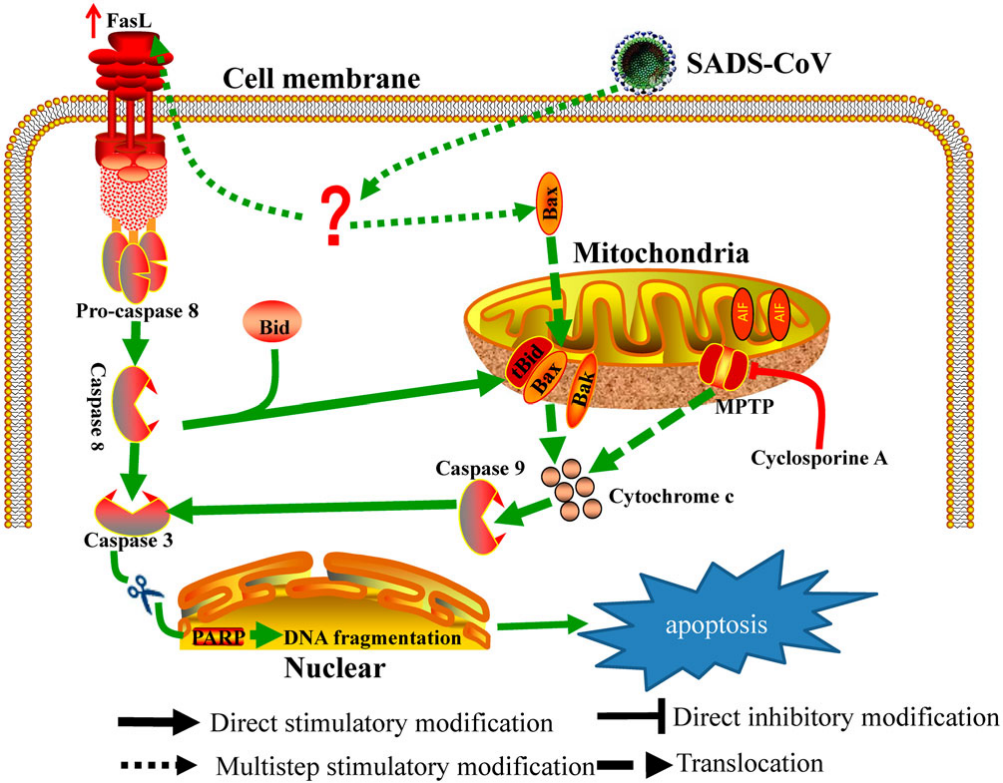
Schematic representation of SADS-CoV-induced apoptosis pathways.

In summary, this study provides insights into the molecular mechanisms of SADS-CoV-induced apoptosis during the course of infection. We demonstrated that (1) SADS-CoV infection causes characteristic morphological and biochemical changes of apoptosis such as DNA fragmentation, chromatin condensation, externalization of phosphatidylserine, caspase activation, and PARP cleavage *in vitro* and *in vivo*; (2) SADS-CoV-induced apoptosis was accompanied by the activation of caspase cascades and promoted effective viral replication *in vitro* during the viral life cycle; (3) The death receptor-mediated extrinsic pathway and mitochondrial-mediated intrinsic pathway are both activated in SADS-CoV-infected cells; (4) The mitochondrial translocation of Bax and concomitant cytosolic release of Cyt c strongly indicate that Bax-mediated MOMP induces intrinsic cell death after SADS-CoV infection; and (5) The life cycle of SADS-CoV is CypD-dependent. Based on our findings, a schematic model of SADS-CoV-induced apoptosis mechanisms is proposed (Figure 8). SADS-CoV is, to our knowledge, the first virus to be reported to trigger direct apoptosis *in vitro* and *in vivo*. Our results provide insights into the comprehensive understanding of the relationship between SADS-CoV and host cells.

## Authors and contributors

Li Feng, Da Shi and Jingyun Ma conceived and designed the experiments; Jiyu Zhang and Yuru Han performed the experiments; Jiyu Zhang, Yuru Han, Hongyan Shi, Jianfei Chen, Xin Zhang, Xiaobo Wang, Ling Zhou, Jianbo Liu, Jialin Zhang, Zhaoyang Ji and Zhaoyang Jing analyzed the data; Jiyu Zhang and Yuru Han revised the manuscript; Li Feng, Da Shi, Jingyun Ma, Jiyu Zhang and Yuru Han wrote the paper.

## Ethics approval

The animal experiments were approved by the Harbin Veterinary Research Institute. The Animal Ethics Committee approval number is Heilongjiang-SYXK-2006-032.
